# Effect of herbicide resistance endowing Ile-1781-Leu and Asp-2078-Gly *ACCase* gene mutations on ACCase kinetics and growth traits in *Lolium rigidum*

**DOI:** 10.1093/jxb/erv248

**Published:** 2015-05-26

**Authors:** Martin M. Vila-Aiub, Qin Yu, Heping Han, Stephen B. Powles

**Affiliations:** ^1^Australian Herbicide Resistance Initiative (AHRI) - School of Plant Biology, University of Western Australia, WA, 6009, Australia; ^2^IFEVA - CONICET - Facultad de Agronomía, Universidad de Buenos Aires (UBA), Buenos Aires, 1417, Argentina

**Keywords:** ACCase activity, *Alopecurus myosuroides*, competition, resistance cost, resistance mutation, RGR.

## Abstract

The rate of herbicide resistance evolution in plants depends on fitness traits endowed by alleles in both the presence and absence (resistance cost) of herbicide selection. The effect of two *Lolium rigidum* spontaneous homozygous target-site resistance-endowing mutations (Ile-1781-Leu, Asp-2078-Gly) on both ACCase activity and various plant growth traits have been investigated here. Relative growth rate (RGR) and components (net assimilation rate, leaf area ratio), resource allocation to different organs, and growth responses in competition with a wheat crop were assessed. Unlike plants carrying the Ile-1781-Leu resistance mutation, plants homozygous for the Asp-2078-Gly mutation exhibited a significantly lower RGR (30%), which translated into lower allocation of biomass to roots, shoots, and leaves, and poor responses to plant competition. Both the negligible and significant growth reductions associated, respectively, with the Ile-1781-Leu and Asp-2078-Gly resistance mutations correlated with their impact on ACCase activity. Whereas the Ile-1781-Leu mutation showed no pleiotropic effects on ACCase kinetics, the Asp-2078-Gly mutation led to a significant reduction in ACCase activity. The impaired growth traits are discussed in the context of resistance costs and the effects of each resistance allele on ACCase activity. Similar effects of these two particular *ACCase* mutations on the ACCase activity of *Alopecurus myosuroides* were also confirmed.

## Highlight

Impaired ACCase enzyme kinetics associated with a particular herbicide resistance mutation is associated with a reduction in plant growth.

## Introduction

Acetyl-coenzyme A carboxylase (ACCase) is a key plant enzyme in lipid biosynthesis and is the target of ACCase-inhibiting herbicides (hereafter referred to as ACCase herbicides). A number of evolved single *ACCase* gene mutations have been identified that confer ACCase herbicide resistance in grass weed species (reviewed in Délye, 2005; Powles and Yu, 2010; Kaundun, 2014). In *Lolium rigidum*, the most widespread crop weed in Australian agriculture, these *ACCase* gene mutations lead to ACCase amino-acid substitutions including Ile-1781-Leu/Val, Trp-1999-Cys/Leu, Ile-2041-Asn/Asp/Thr/Val, Asp-2078-Gly, Cys-2088-Arg/Phe, and Gly-2096-Ala (Yu *et al.*, 2007b; Malone *et al.*, 2013). Molecular and survey analyses have indicated that the frequency and distribution of these evolved mutant *ACCase* alleles vary among populations (Yu *et al.*, 2007b; Malone *et al.*, 2013; Vila-Aiub *et al.*, 2009b; Keshtkar *et al.*, 2015).

The crystal structures of the ACCase carboxyl-transferase domain of yeast ACCase revealed that ACCase herbicides are bound in the carboxyl-transferase active site (Zhang *et al.*, 2004). Therefore, specific *ACCase* gene mutations, while conferring herbicide resistance, may also have an impact on ACCase activity (Délye *et al.*, 2005). For example, the Asp-2078-Gly mutation confers a high-level resistance (*I*_*50*,_ as compared to the susceptible counterpart) to diclofop-methyl, haloxyfop, and tralkoxydim, and the specific ACCase activity (in absence of herbicide treatment) is significantly reduced (Yu *et al.*, 2007b).

Herbicide target-site resistance mutations can incur resistance costs due to impaired enzyme catalytic capacity and ⁄or reduced substrate affinity, and/or altered feedback inhibition resulting in insufficient or excessive product biosynthesis (Purrington and Bergelson, 1999; Vila-Aiub *et al.*, 2009b; Yu *et al.*, 2010). From an ecological evolutionary context, rapid herbicide resistance evolution is possible if the gene mutation provides a significant level of resistance and yet show no or negligible fitness cost (the magnitude of such a cost is assessed in the absence of herbicide selection) (Vila-Aiub *et al.*, 2015). Conversely, genetic traits endowing a high or low resistance level and with a fitness cost are likely to evolve relatively slowly.

Here, the pleiotropic effects of the *ACCase* gene Ile-1781-Leu and Asp-2078-Gly mutations on both ACCase activity and kinetics and fitness-related growth traits were assessed in *L. rigidum*. This study provides a better understanding of the biochemical basis of resistance cost and evolutionary dynamics of ACCase herbicide resistance alleles in *L. rigidum*.

## Materials and methods

### Plant material and ACCase herbicide resistance gene mutations

A number of field-evolved ACCase herbicide–resistant *L. rigidum* populations collected across the WA wheatbelt (Owen *et al.*, 2007) were subjected to detailed molecular characterization enabling identification of populations resistant to ACCase herbicides owing to specific *ACCase* gene mutations (Yu *et al.*, 2007b). Purified populations that were each homozygous (*RR*) for the specific ACCase herbicide resistance mutations Ile-1781-Leu and Asp-2078-Gly were generated and fully characterized (Yu *et al.*, 2007b). This was achieved by identifying (via sequencing and PCR-based marker analysis) plants homozygous for the specific mutation, and growing these homozygous plants to maturity and allowing cross pollination in pollen-proof cages to produce seeds for one generation. Homozygosity of the progeny plants for the specific *ACCase* gene mutation in each purified population was confirmed using PCR-based marker analysis as described in Yu *et al.* (2007b). The bulk-crossing progeny of the two purified resistant populations were used in all subsequent experiments. This experimental approach precludes any confounding effects of potential differences in resistance costs attributed to the dominance of the cost (*RR* vs. *RS*) (Roux *et al.*, 2004). Four herbicide-susceptible *L. rigidum* populations (H3/6, H4/6, H4/33, and VLR1, hereinafter called S_1_, S_2_, S_3_, and S_4_, respectively) exhibiting susceptible ACCase (Yu *et al.*, 2007b; Yu *et al.*, 2009) were used as wild-type controls to minimize differences in genetic background between the ACCase herbicide–resistant and various susceptible populations (Vila-Aiub *et al.*, 2009b; Vila-Aiub *et al.*, 2011). This experimental approach assumes that a statistically significant difference in mean trait values between compared resistant and susceptible populations indicates that those differences are likely caused by pleiotropic effects of resistance gene(s) (Cousens *et al.*, 1997; Strauss *et al.*, 2002). The methodological approaches used in these studies enable the independent comparison of each ACCase herbicide–resistant population versus all ACCase herbicide–susceptible populations. Information on the six *L. rigidum* populations used in this study is in Supplementary Table S1.

To correlate the effect of the specific ACCase herbicide resistance mutations (Ile-1781-Leu or Asp-2078-Gly) on the expression of resistance costs at both the whole plant level and enzyme level, ACCase activity and kinetics associated with each resistance mutation were evaluated. Individuals from the susceptible S_4_ population and two other *L. rigidum* populations collected from WA cropping systems (WALR60, WALR70) with known susceptible ACCase sequences served as wild-type controls to measure ACCase activity and kinetics (Yu *et al.*, 2007a; Yu *et al.*, 2009). As fitness costs associated with the Asp-2078-Gly mutation but not with the Ile-1781-Leu mutation have been reported previously in *Alopecurus myosuroides* (Menchari *et al.*, 2008), the effect of these two resistance mutations on ACCase activity was therefore also assessed in *A. myosuroides*. A purified *A. myosuroides* population homozygous for the Asp-2078-Gly mutation (Délye *et al.*, 2003; Délye *et al.*, 2005) and a field population containing 92% plants homozygous for the Ile-1781-Leu mutation were used in this study (Délye, personal communications). Two ACCase herbicide–susceptible *A. myosuroides* populations were used as wild-type controls.

### ACCase activity and kinetics associated with specific ACCase herbicide resistance mutations

Individuals homozygous for each specific ACCase resistance mutation (Ile-1781-Leu or Asp-2078-Gly) and susceptible individuals were grown in glasshouse conditions (see details below). At the three- to four-leaf stage, the above-ground leaf material (about 3g) was harvested at soil level from each population (at least 30–40 seedlings per harvest), snap-frozen in liquid nitrogen, and stored at −80°C. The ACCase *in vitro* assay was conducted according to the method of Yu *et al.* (2004) with modifications. The frozen material was ground to a fine powder with a mortar and pestle in liquid nitrogen and homogenized in 10mL extraction buffer containing 100mM Tris (pH 8.0), 1mM EDTA, 10% (v/v) glycerol, 2mM isoascorbic acid, 1mM PMSF, 0.5% PVP-40, 0.5% insoluble PVP, and 20mM DTT. The homogenate was centrifuged at 27 000 *g* for 15min. The supernatant was brought to 40% (NH_4_)_2_SO_4_ saturation by drop-wise addition of saturated (NH_4_)_2_SO_4_, and stirred for 10min. The solution was centrifuged at 27 000 *g* for 20min. The pellet was resuspended in 1.5mL elution buffer (50mM Tricine, pH 8.0, 2.5mM MgCl_2_, 50mM KCl, 1mM DTT) and desalted on a Sephadex G-25 column pre-equilibrated with elution buffer. Protein concentration of each desalted sample was determined (Bradford, 1976) and the sample was immediately used for assay.

ACCase activity was determined by quantifying the incorporation of NaH^14^CO_3_ into acid-stable product malonyl-CoA. The enzyme extract was incubated at 30°C in reaction mixture that contained 10mM Tricine-KOH, pH 8.3, 5mM ATP, 10mM MgCl_2_, 0.1% BSA, 2.5mM DTT, and 10mM NaHCO_3_ (supplemented with an average of 24 kBq NaH^14^CO_3_, 2.18 GBq mmol^−1^). The reaction was started by the addition of acetyl-CoA at a final concentration of 0.5mM and was stopped after 10min by the addition of concentrated HCl. Assays without acetyl-CoA were used as controls. Acid stable radioactivity was measured by a scintillation counter and ACCase activity was calculated using the isotope dilution method. For ACCase kinetic measurement (only conducted for *L. rigidum*), 40–120 µg protein was used, as this protein level catalyses a linear rate of malonyl-CoA formation under these experimental conditions. The highest concentration of 1.5, 3, and 20mM of acetyl-CoA, ATP, and HCO_3_ was respectively used in the determination of *Km* values. Total protein was normalized to 100 µg for all sample to measure ACCase-specific activity.

*Km* values were calculated using a non-linear regression analysis by fitting the data to the Michaelis–Menten equation *ν* = *V*S / (*Km* + S), where S is the concentration of the substrate pyruvate, *ν* is the reaction velocity at any pyruvate concentration, and *V* is the maximal reaction velocity (*Vmax*). Each assay contained two technical replicates and four independent enzyme extracts were used for each assay set. Data were subjected to ANOVA using SAS Software (Version 9.3, Cary, NC, SAS Institute Inc. 2002–2010). Means were separated using Fisher’s protected least significant difference (LSD) test at the 5% level of probability.

### Resistance cost associated with specific *ACCase* gene resistance mutations

Experiments designed to assess growth in both isolated plants (i.e. no competition) and under interspecific competition were conducted twice. Relative growth rate (RGR) and components [net assimilation rate (NAR), leaf area ratio (LAR)], and resource allocation to roots, stems, and leaf area were estimated in isolated plants growing without competition. Competitive responses of *L. rigidum* expressing specific ACCase herbicide resistance mutations (Ile-1781-Leu or Asp-2078-Gly) and susceptible-wild type were estimated in competition with wheat (*Triticum aestivum*). Both RGR and resource competitive responses are useful eco-physiological parameters to denote the expression of herbicide-resistance costs, as variations in these traits are positively correlated with variations in plant competitive and establishment ability, and fecundity (Grime and Hunt, 1975; Goldberg, 1990; Weiner, 2004; Vila-Aiub *et al.*, 2005).

### Growth of isolated plants without competition

Seeds of the homozygous ACCase herbicide–resistant mutant (Ile-1781-Leu, Asp-2078-Gly) and susceptible wild-type genotypes (S_1_ to S_4_) were germinated on 0.7% (w/v) agar (12h in light at 25°C, 12h dark at 15°C). After 4 days, individual seedlings (2cm height) were transplanted into individual pots (9cm diameter, 13cm height) containing a standard potting mix (50% peatmoss and 50% river sand). Plants were grown in a glasshouse, arranged in a completely randomized design. Pots were regularly re-arranged to randomize any environmental differences within the glasshouse. Mean growing temperature conditions fluctuated between 20°C (day) and 15°C (night), near optimum for this species. Plants were harvested 10 and 24 days after transplanting. Above-ground and root biomass and leaf area were estimated in each harvest. Plants were removed from soil and roots were washed with tap water. Leaf area per plant was determined with a digital leaf area meter (LI-3100; LiCor, Lincoln, NE, USA). Above-ground material (shoots were divided into leaf material and stems including the leaf sheath) and roots were oven-dried at 80°C for 72h, and dry biomass recorded. RGR and its components (NAR and LAR) were calculated for each treatment combination (genotype × harvest). There were 20 replicates per treatment (seven genotypes × two harvest times). Plants were watered regularly and fertilized weekly with a liquid fertilizer [N 19% (NH_2_ 15%, NH_4_ 1.9%, NO_3_ 2.1%), P 8%, K 16%, Mg 1.2%, S 3.8%, Fe 400 ppm, Mn 200 ppm, Zn 200 ppm, Cu 100 ppm, B 100 ppm, and Mo 10 ppm].

A software program (Hunt *et al.*, 2002) was used to calculate growth parameters, which are derived according to classical growth analysis (Hunt, 1982; Poorter and Nagel, 2000). The unbiased formula proposed by Hoffmann and Poorter (2002) was used to calculate RGR. The variance (σ^2^ or *V*) associated with RGR was estimated with Causton and Venus’ formula (1981). The degrees of freedom associated with RGR and its components were *n* – 2, where *n* was the total number of plants used in two harvests. One-way ANOVA was performed to compare RGR and its components for ACCase resistance and susceptible wild-type *L. rigidum* genotypes. Dunnett’s post-hoc test was used to compare mean values of the ACCase herbicide resistance genotypes against the susceptible wild-type reference genotypes (α = 5%).

Resistance cost associated with each of the ACCase herbicide resistance mutations was estimated as per Vila-Aiub *et al.* (2009b):

$$RC_{\left(R/S\right)}\left(\%\right)=\left[1-\left(\frac{r_{R}}{r_{S}}\right)\right]*100$$ (1)

where *RC*_(*R/S*)_ represents the resistance cost (*RC*) of the herbicide-resistant (*R*) genotype relative to the herbicide-susceptible (*S*) genotype, $r_{R}$ denotes the response of the resistant genotype *R*, and $r_{S}$ is the response of the susceptible wild-type genotype *S*.

### Growth of plants under competition

A target-neighbourhood experimental design was employed to evaluate resource competitive responses of the homozygous ACCase herbicide–resistant mutants (Ile-1781-Leu, Asp-2078-Gly) and susceptible wild-type genotypes (S_1_ to S_4_) grown in competition with wheat (Supplementary Fig. S1) (Gibson *et al.*, 1999). Competitive responses to environmental resources are related to a plant’s ability to persist regardless of the presence of a competitor (wheat). Thus, the vegetative performance of the target plants (i.e. ACCase *RR* or *SS* genotype) was evaluated under increasing densities and biomass of neighbour wheat plants (Weiner, 1982; Goldberg and Werner, 1983).

Assessment of competitive responses of target ACCase *RR* and *SS* target plants was conducted under size-asymmetric competition from wheat (Goldberg, 1990). Wheat (‘Wyalkatchem’ cv) was seeded in pots (25cm diameter × 23cm height) containing potting mix (50% peatmoss and 50% river sand) according to the planting patterns (Supplementary Fig. S1). Seeds of uniform weight of the ACCase *RR* and *SS* genotypes were germinated as described before. When the wheat was at the three-expanded-leaf stage (15cm height), one-leaf stage *L. rigidum* seedlings were transplanted (2cm height) into the wheat-containing pots. A slow-release fertilizer (Macrocote Blue Plus) (12g/pot) [w/w N 16%, (NH_2_ 8.4%, NH_4_ 6.45%, NO_3_ 1.47%), P 4%, K 10%, S 5%, Mg 0.63%, Fe 0.20%, Cu 0.03%, Zn 0.03%, and Mn 0.08%] and liquid fertilizer [N 19% (NH_2_ 15%, NH_4_ 1.9%, NO_3_ 2.1%), P 8%, K 16%, Mg 1.2%, S 3.8%, Fe 400 ppm, Mn 200 ppm, Zn 200 ppm, Cu 100 ppm, B 100 ppm, and Mo 10 ppm] were applied during the tillering phase. Pots were well-watered at all times and liquid urea (46% N) was applied regularly. Experimental units were arranged in a completely randomized design and placed outdoors under prevailing field conditions during the normal winter growing season for *L. rigidum*.

After 2 months of growth, above-ground vegetative biomass and leaf area of individual target plants for each corresponding ACCase *RR* or *SS* genotype were determined as above. Above-ground biomass of neighbour wheat plants was evaluated. Each experimental treatment had seven replicates.

To standardize for differences in productivity, data for biomass production and leaf area of target plants in the presence of neighbours were expressed as a percentage of that trait in the absence of competition (Goldberg and Scheiner, 2001). Per capita and unit-size competitive responses were analysed using a hyperbolic non-linear model to describe the response of the target plants to increasing density and biomass of neighbour wheat plants (Weiner, 1982; Goldberg and Werner, 1983; Goldberg and Fleetwood, 1987):

$$G=\;\frac{a}{1+bx}$$ (2)

where *G* represents the fitness trait (biomass or leaf area) of the target plant at neighbour density or biomass *x*, *a* is the fitness trait of the target plant in the absence of competitors (neighbours) (*x* = 0), and *b* the slope of the regression. The model was fitted by least-squares regression analysis using SigmaPlot software (version 12.0; Systat Software Inc.). The variance in growth of the target plant explained by the density or biomass of neighbours (*R* ^2^ of the regression model) indicates the importance of resource competition relative to other factors affecting target plant performance (Goldberg and Fleetwood, 1987).

The growth response of target plants (either with ACCase herbicide resistance or wild-type alleles) to both increasing number (i.e. per capita response) and overall biomass (i.e. per unit-size size) of neighbour wheat plants was established after comparison of regression slopes (*b* parameter) by one-way ANOVA. Lower and higher slopes denote strong and weak competitive responses, respectively (Weiner, 1982; Goldberg and Werner, 1983; Goldberg, 1987). The hyperbolic model was fitted after log-transformation of data (*y* = log [*x*]) to comply with regression analysis assumptions.

## Results

### Effects of ACCase herbicide resistance mutations on ACCase activity and kinetics

**Fig. 1.**
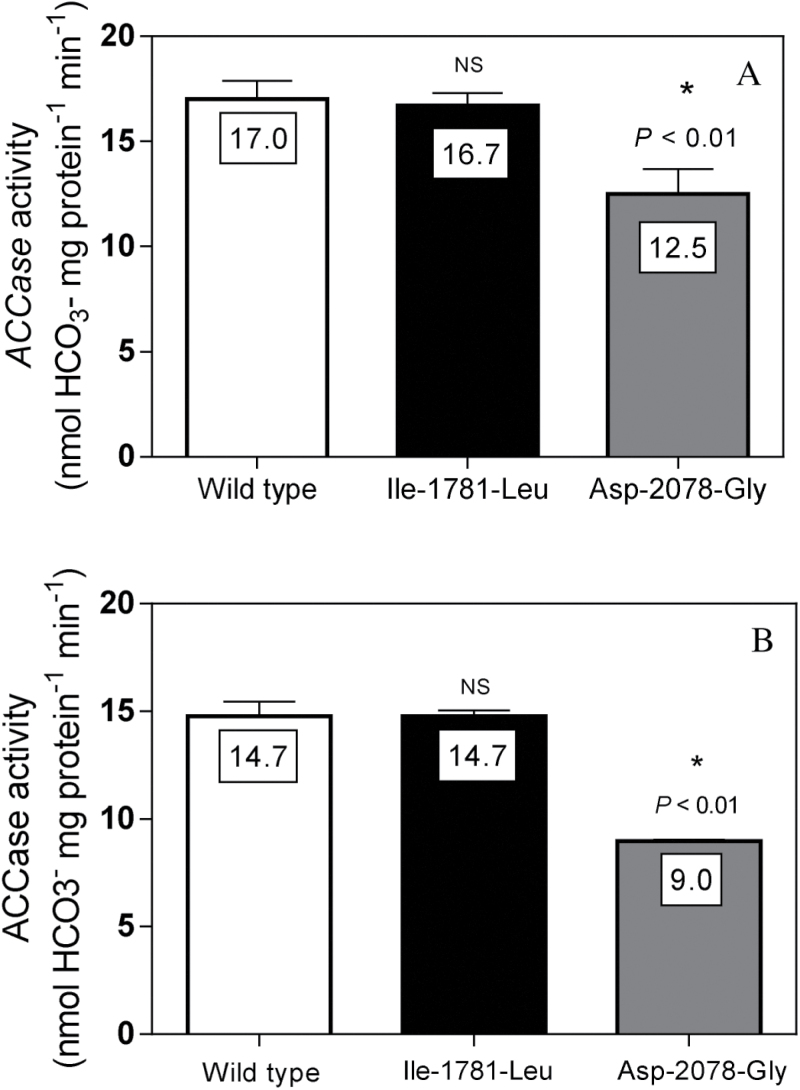
Extractable ACCase activity measured from partially purified enzyme extracts from the three- to four-leaf stage (A) *L. rigidum* and (B) *A. myosuroides* plants of ACCase herbicide–susceptible (*SS*), wild type (white bar), and homozygous resistant (*RR*) mutants (Ile-1781-Leu and Asp-2078-Gly). Asterisks indicate significant differences between mean values according to Dunnett’s post-hoc test using the mean ACCase herbicide-susceptible populations as control (α = 0.05). NS: not significant.

The extractable ACCase activity was the same in *L. rigidum* plants homozygous for the Ile-1781-Leu mutation as in the wild-type herbicide-susceptible plants (Fig. 1A). In contrast, a significantly lower specific ACCase activity was found in plants homozygous for the Asp-2078-Gly (Fig. 1A). Plants with the Asp-2078-Gly mutation showed only 70% extractable ACCase activity (specific activity) when compared to susceptible wild-type plants. Similarly in *A. myosuroides*, plants homozygous for the Asp-2078-Gly mutation displayed a significantly reduced (40%) ACCase activity, whereas plants with the Ile-1781-Leu mutation showed no change in ACCase activity, relative to the two susceptible *A. myosuroides* populations (Fig. 1B).

The ACCase substrate affinity (*Km*) for acetyl-CoA, HCO_3_^-^, and ATP were determined for the two resistance mutations and compared to the wild-type ACCase controls. Neither of the two *ACCase* mutations changed *Km* values, especially for acetyl-CoA, although the Ile-1781-Leu mutation increased *Km* (ATP) (Table 1). Calculated *Vmax* under each substrate also revealed unchanged maximum reaction velocity for the Ile-1781-Leu mutants, despite significant reduced values for the Asp-2078-Gly mutants (33–48%) (Table 1).

**Table 1.** *Apparent* Km *(mM) and* Vmax *(nmol HCO_3_^−^ mg protein^−1^ min^−1^) values for acetyl-CoA, HCO_3_^−^, and ATP substrates determined for partially purified ACCase from plants homozygous for Ile-1781-Leu, Asp-2078-Gly, and susceptible wild type in* L. rigidum

	Acetyl-CoA		HCO_3_^−^		ATP	
*ACCase* resistance mutations	*Km*	*Vmax*	*Km*	*Vmax*	*Km*	*Vmax*
Wild type	0.091_a_	35.6_a_	3.5_a_	38.0_a_	0.116_a_	38.1_a_
Ile-1781-Leu	0.094_a_	34.7_a_	3.5_a_	33.5_ab_	**0.158**_**b**_	35.0_a_
Asp-2078-Gly	0.079_a_	**18.4**_**b**_	3.3_a_	**25.6**_**b**_	0.097_a_	**20.4**_**b**_

ACCase kinetics parameters corresponding to the susceptible wild-type allele are averaged values from individuals of the three ACCase herbicide–susceptible populations (S_1_, WALR60, WALR70). Means with different letters within a column are significantly different according to Fisher LSD test (α = 0.05).

### Examination for resistance costs

#### Growth of isolated plants without competition

**Fig. 2.**
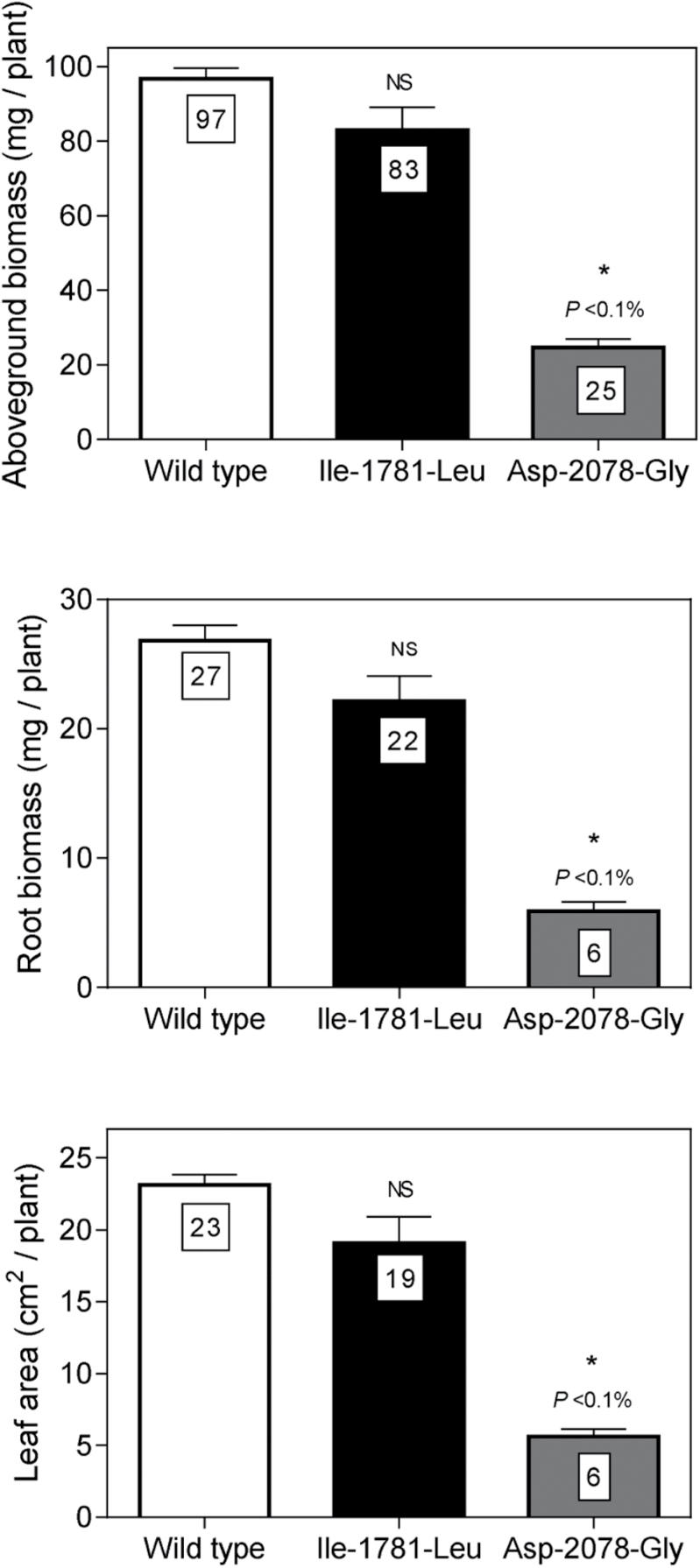
Above-ground and root biomass and leaf area produced by *L. rigidum* plants of susceptible wild-type *ACCase* (*SS*) (white bar) and homozygous resistant (*RR*) (Ile-1781-Leu, Asp-2078-Gly) mutants after 24 days of growth in the absence of plant resource competition. Growth traits corresponding to plants carrying the ACCase herbicide–susceptible alleles are mean estimates resulting from all susceptible wild type populations (S_1_, S_2_, S_3_ and S_4_). Vertical bars denote SE of the mean (n = 15). Asterisks indicate significant differences between mean values according to Dunnett’s post-hoc test using the mean ACCase herbicide-susceptible populations as control (α = 0.05). NS: not significant.

Estimated RGR, NAR, and LAR parameters did not differ among the four ACCase herbicide–susceptible populations (*P* > 0.05) (Table 2). Therefore, a mean growth parameter was calculated for all susceptible populations and used as a reference estimate for further comparisons. Growth analysis revealed that plants homozygous for the Ile-1781-Leu mutation exhibited similar RGR, NAR, and NAR growth parameters to herbicide-susceptible plants (Table 2). At the end of the growing period, the Ile-1781-Leu mutants showed above-ground and root biomass and leaf area similar to susceptible wild-type plants (Fig. 2).

**Table 2.** *Mean estimates of RGR and components NAR and LAR associated with* L. rigidum *genotypes exhibiting wild-type ACCase and specific homozygous (*RR*) resistance mutants (Ile-1781-Leu, Asp-2078-Gly)*

*ACCase* gene mutation	RGR (day^−1^)	NAR (g cm^−2^ day^−1^)	LAR (cm^2^ g^−1^)
Wild type (S_1_)	0.22 (0.004)	0.0012 (3E−05)	178 (5)
Wild type (S_2_)	0.24 (0.004)	0.0013 (4E−05)	197 (6)
Wild type (S_3_)	0.24 (0.004)	0.0013 (3E−05)	193 (8)
Wild type (S_4_)	0.24 (0.005)	0.0013 (5E−05)	188 (8)
**Wild type** (pooled S_1_–S_4_)	**0.23** ^**a**^ **(0.004)**	**0.0013** ^**a**^ **(2E−05)**	**189** ^**a**^ **(4)**
Ile-1781-Leu	0.21^a^ (0.005)	0.0012^a^ (4E−04)	180^a^ (6)
Asp-2078-Gly	**0.16** ^**b**^ (0.007)	**0.008** ^**b**^ (4E−04)	190^a^ (11)

Growth was estimated in isolated plants in absence of herbicide selection for a period of 24 days since seed germination. Comparison of RGR and components were conducted between each specific *ACCase* gene mutation and pooled wild-type populations (S_1_–S_4_). Numbers in parenthesis denote standard error of estimates. Different superscript letters indicate significant differences in mean estimates according to Dunnett’s test (α = 0.05). Numbers in brackets denote standard error of the mean (n = 40).

Homozygous Asp-2078-Gly mutants exhibited significantly reduced RGR, driven by reductions in NAR but not LAR (Table 2), hence expressing a higher resistance cost of 30% and 38% associated with RGR and NAR, respectively (Table 2). Reduced RGR estimates associated with plants homozygous for the Asp-2078-Gly mutation accounted for the lower above-ground (74% reduction), root biomass (78% reduction), and leaf area (74% reduction) produced compared to the susceptible individuals (Fig. 2).

#### Growth of plants under competition

The hyperbolic model adequately explained variations in growth responses (i.e. biomass, leaf area) of target plants to increasing densities (per capita response) and biomass (per unit-size response) of neighbouring wheat plants (*R* ^2^ = 0.64 – 0.91, *P* < 0.0001). Competitive responses (i.e. ability to persist and produce biomass in the presence of competitors) of plants associated with the two specific ACCase herbicide resistance mutations were evaluated by comparing estimates of regression slopes in the presence of wheat: the steeper the slope (higher value) the weaker the competitive response. As the observed per capita and per-unit size based competitive responses of target plants were similar, only the latter are shown. Competitive responses of target *L. rigidum* plants to wheat varied depending on the specific ACCase herbicide resistance mutation expressed by plants.

**Fig. 3.**
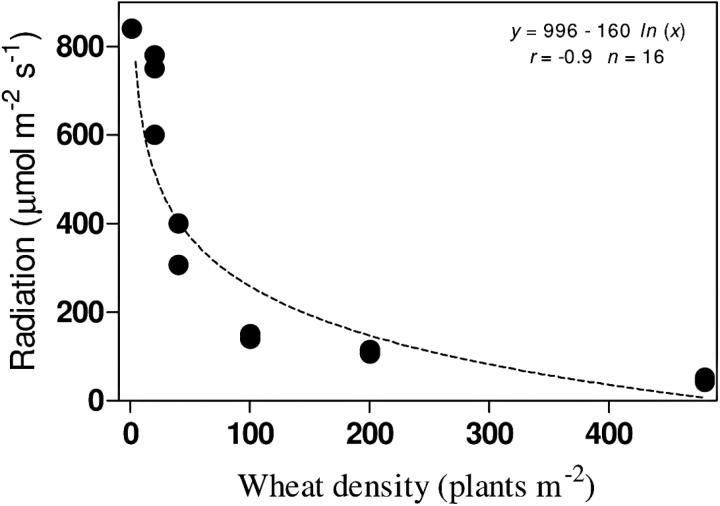
Photosynthetically active radiation (µmol m^−2^ s^−1^) intercepted by target *ACCase* mutants (1781-Leu, 2078-Gly) and susceptible wild-type plants as a function of increasing wheat density (0–600 plants m^–2^). Estimations were performed 37 days after the start of the experiment.

**Fig. 4.**
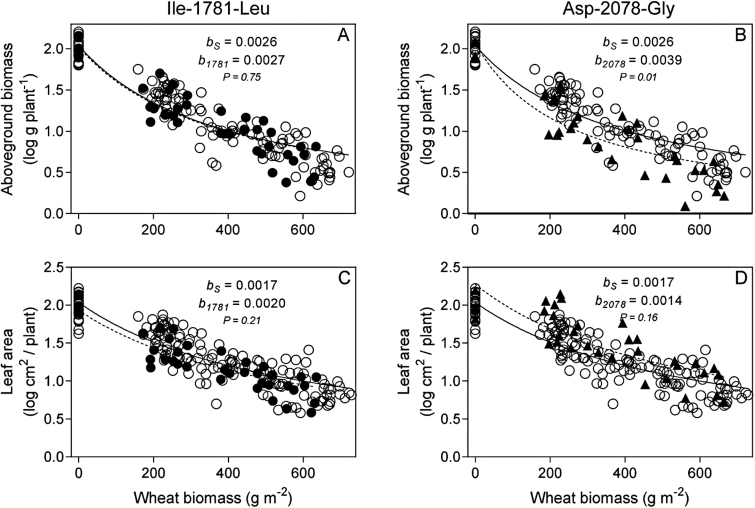
Variations in above-ground vegetative biomass and leaf area of target ACCase herbicide–resistant mutants (*RR*) Ile-1781-Leu (●) and Asp-2078-Gly (▲) and susceptible wild-type (*SS*) (○) individuals (S_1_, S_2_, S_3_, and S_4_) under increasing biomass of wheat neighbour plants. Comparison of regression slopes (*b* parameter) determines hierarchies in per unit-size competitive responses of target plants to wheat neighbour plants.

As expected, an increase in wheat density negatively correlated with the intercepted radiation by target *L. rigidum* plants (Fig. 3). This led to a reduction in the size of the *L. rigidum* plants with increasing wheat competition (Fig. 4). However, as evident from the slopes of the regressions, reductions in both aerial biomass and leaf area of plants homozygous for the Ile-1781-Leu mutation were similar to those shown by the herbicide-susceptible wild-type plants (Fig. 4A-C).

In comparison to the biomass attained by herbicide-susceptible target plants, the Asp-2078-Gly mutants produced concomitantly less biomass with increasing wheat competition (Fig. 4B-D). This was denoted by steeper slopes estimated after regression. The reduction in size of target plants of the Asp-2078-Gly mutation was not accompanied by a reduction in leaf area, as for the ACCase herbicide–susceptible individuals (Fig. 4D). An extra indication of the weak competitive response (i.e. above-ground biomass) associated with the Asp-2078-Gly resistance mutation was indicated by mortality of four target plants when competing with the crop at 200 and 480 wheat plants m^−2^.

## Discussion

The dynamics of herbicide resistance evolution in plants is dependent on fitness traits endowed by herbicide resistance mutations both in the presence and absence of herbicide selection (Jasieniuk *et al.*, 1996). Resistance costs associated with herbicide resistance alleles account for the likelihood of fixation of novel resistance mutations in herbicide-unselected weed populations or populations in which selection has been discontinued.

In annual species like *L. rigidum*, changes in growth rate and plant size correlate positively with changes in reproductive traits (Bazzaz *et al.*, 1987; Weiner, 2004; Vila-Aiub *et al.*, 2009a; Weiner *et al.*, 2009). The present study assessed various growth traits defining the overall population fitness associated with two specific ACCase herbicide resistance gene mutations in *L. rigidum*. The correlation between the impact of these mutations on ACCase activity and kinetics and those fitness components was also examined. Comparisons were made between homozygous plants with either the *ACCase* Ile-1781-Leu or Asp-2078-Gly mutations and susceptible wild-type plants from four different *L. rigidum* populations.

The experimental results reveal that (i) both the Ile-1781-Leu and Asp-2078-Gly resistance mutations may endow resistance to ACCase herbicides without significant interference on normal substrate binding (*Km*), (ii) the levels of ACCase activity and catalytic capacity (*Vmax*) associated with each mutation may be correlated with the magnitude of resistance costs, and (iii) resistance costs are associated with the Asp-2078-Gly mutation but not with the Ile-1781-Leu mutation.

### *ACCase* Ile-1781-Leu mutation: no impact on ACCase kinetics and no resistance cost

Homozygous resistant Ile-1781-Leu mutants had similar RGR-NAR and resource competitive responses compared to herbicide-susceptible plants possessing the wild-type ACCase. These results are in agreement with two previous studies showing negligible fitness costs associated with the Ile-1781-Leu mutation in *L. rigidum* and *A. myosuroides* (Vila-Aiub *et al.*, 2005; Menchari *et al.*, 2008). When introgressed into *Setaria italica*, the Ile-1781-Leu mutants even showed a fitness advantage in the absence of herbicide selection (Wang *et al.*, 2010). It is likely that the absence of resistance cost associated with this Ile-1781-Leu mutation in *L. rigidum* is because there is no adverse effect of this mutation on ACCase enzyme kinetics. The Ile-1781-Leu substrate affinity (*Km*) and velocity of product formation (*Vmax*) were similar to the susceptible wild-type ACCase (Vila-Aiub *et al.*, 2009b; Yu *et al.*, 2010). *A. myosuroides* plants also showed no adverse effects of the Ile-1781-Leu mutation on ACCase activity (Fig. 1B).

### *ACCase* Asp-2078-Gly resistance mutation: impact on ACCase kinetics and resistance cost

Plants homozygous for the Asp-2078-Gly mutation exhibited reduced RGR-NAR growth parameters, implying a negative association with the plant’s efficiency in capturing light, assimilating CO_2_, and/or storing photoassimilates. Significant reductions in leaf area and above-ground and root biomass were found in individuals possessing the Asp-2078-Gly mutation. When under competition, plants with the Asp-2078-Gly mutation also showed impaired growth under resource competition evident as a weak competitive response to wheat when compared to plants with the susceptible wild-type ACCase. Weak competitive responses of plants with the Asp-2078-Gly mutation were consistent with both increasing wheat density and biomass. This result indicates the presence of plant traits associated with the Asp-2078-Gly mutation other than ‘reduced plant size’ in contributing to the weak competitive response, as differences in competitive responses resulting only from different plant sizes should not be apparent when adjusted by size (neighbour weight) (Goldberg and Scheiner, 2001). In competitive conditions in which light becomes the most limiting resource [i.e. only 6–13% available photosynthetically active radiation was observed at high wheat densities (200–480 plants m^−2^)], plants displaying reduced NAR are expected to make inefficient use of radiation.

Given that plant reproductive effort is a function of RGR and the amount of resources allocated to vegetative biomass, the observed reduction in size associated with the Asp-2078-Gly resistance mutation in plants grown without and with competition are likely to translate into reductions in fecundity compared to individuals with the wild-type ACCase (Bazzaz *et al.*, 1987; Weiner, 2004; Weiner *et al.*, 2009). This is especially true for annual species like *L. rigidum* in which reduced plant sizes and RGR correlate positively with reduced reproductive traits (Vila-Aiub *et al.*, 2009a).

It is clear that the Asp-2078-Gly resistance mutation renders ACCase resistant to ACCase herbicides. This mutation does not change ACCase substrate affinity (*Km*) but there is a nearly 50% reduction in the ACCase velocity (*Vmax*) to catalyse the formation of malonyl-CoA at the expense of natural substrates (acetyl-CoA, ATP, and HCO_3_). The overall reduction in ACCase-specific activity (30%) and *Vmax* may lead to shortage of lipids available for rapid growth and be correlated with the impaired growth responses observed in plants homozygous for the *ACCase* Asp-2078-Gly mutation.

The Asp-2078-Gly mutation in *A. myosuroides* has also been associated with a resistance cost (Menchari *et al.*, 2008). As demonstrated here (Fig. 1B), a plausible explanation for this reduced fitness is the impaired ACCase activity associated with this particular target site mutation.

### Insights into the evolution of ACCase herbicide resistance

Conditions favouring the rapid evolution and fixation of novel herbicide target site resistance alleles in weed populations include both negligible resistance costs (*RC*, see equation 1) manifested when the herbicide selection is relaxed and a significant fitness advantage or plant protection under herbicide selection (Vila-Aiub *et al.*, 2009b). Previous studies have characterized high levels of resistance to some aryloxyphenoxypropionate and cyclohexanedione ACCase herbicides endowed by the Ile-1781-Leu and Asp-2078-Gly mutations in various species (Délye *et al.*, 2005; Délye *et al.*, 2008; Yu *et al.*, 2007b).

In the absence of herbicide selection, the lack of resistance costs suggests that no apparent constraints exist for the ACCase Ile-1781-Leu resistance allele to persist and be fixed in populations. Empirical evidence shows that the Ile-1781-Leu resistance allele has been fixed in some grass species and found in ancient *A. myosuroides* individuals never exposed to herbicides (Délye and Michel, 2005; Délye *et al.*, 2013). On the contrary, it is predicted that the environment will pose more limits for the ACCase Asp-2078-Gly resistance allele to sustain once herbicide selection is relaxed. Thus, under herbicide selection, it is expected that both the Ile-1781-Leu and Asp-2078-Gly mutations increase their population frequency. Once selection is discontinued, the Ile-1781-Leu mutation is more likely to persist than the Asp-2078-Gly mutation.

## Supplementary data

Supplementary data are available at *JXB* online.

Supplementary Table S1. Information on the *L. rigidum* populations used in the study.

Supplementary Fig. S1. Design of the plant resource competition experiment.

Supplementary Data
