# Supplementary material for: Effect of herbicide resistance endowing Ile-1781-Leu and Asp-2078-Gly ACCase gene mutations on ACCase kinetics and growth traits in Lolium rigidum
Source: J Exp Bot. 2015 May 26;66(15):4711–8. doi: 10.1093/jxb/erv248 (PMC4507778; doi:10.1093/jxb/erv248)
Supplement: Supplementary Data [file supp_66_15_4711__index.html]

Effect of herbicide resistance endowing Ile-1781-Leu and Asp-2078-Gly ACCase gene mutations on ACCase kinetics and growth traits in Lolium rigidum — Effect of herbicide resistance endowing Ile-1781-Leu and Asp-2078-Gly ACCase gene mutations on ACCase kinetics and growth traits in Lolium rigidum — Supplementary Data 

# Effect of herbicide resistance endowing Ile-1781-Leu and Asp-2078-Gly *ACCase* gene mutations on ACCase kinetics and growth traits in *Lolium rigidum*

## Supplementary Data

Data files

- Supplementary Data - Supplementary Data
