# Supplementary material for: Effect of herbicide resistance endowing Ile-1781-Leu and Asp-2078-Gly ACCase gene mutations on ACCase kinetics and growth traits in Lolium rigidum
Source: J Exp Bot. 2015 May 26;66(15):4711–8. doi: 10.1093/jxb/erv248 (PMC4507778; doi:10.1093/jxb/erv248)

Table 1 Information on the *Lolium rigidum* populations used and their response to two *ACCase*- inhibiting herbicides (diclofop-methyl, sethoxydim). Seedlings were treated at the 2- to 3-leaf stage and mortality was determined 21 day after treatment. *SS*: homozygous herbicide susceptible; *RR*: homozygous herbicide resistant

ACCase mutation	Original population	Plant survival (%)		
		Diclofop-methyl	Sethoxydim	
		1000 g ha^{-1}	60 g ha^{-1}	200 g ha^{-1}
Wild type (*SS*)	H3/6	0	0	0
Wild type (*SS*)	H4/6	0	0	0
Wild type (*SS*)	H4/33	0	0	0
Wild type (*SS*)	VLR1	0	0	0
Ile-1781-Leu (*RR*)	M1/25	100	100	100
Asp-2078-Gly (*RR*)	M3/4	100	100	100

Fig. 1 Overview of experimental design (target-neighbourhood model) to assess plant responses associated with *ACCase* herbicide resistance (*RR*) (1781-Leu and 2078-Gly) and susceptible (*SS*, wild type) (S_1, S_2, S_3 and S_4) alleles to competition with a crop (wheat) at the vegetative plant stage. Closed (●) symbols represent individual target plants (*RR* or *SS*). Open (○) symbols represent neighbour plants (wheat) at increasing densities.

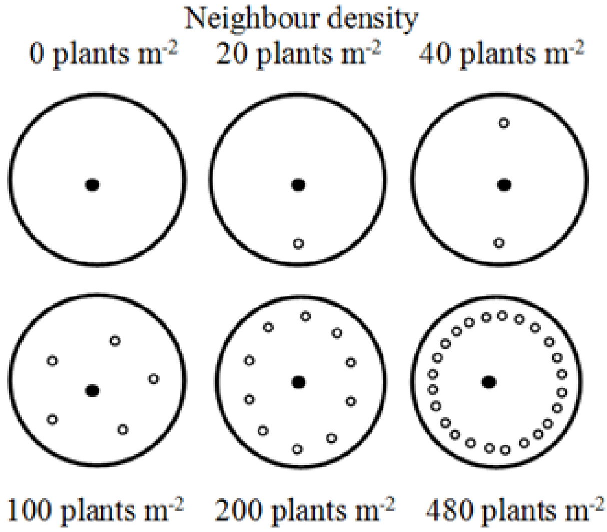

Supplement: Supplementary Data [file supp_erv248_jexbot145730_file001.pdf]
